# Incidence and Factors Associated with Self-Reported Skin Symptoms of Allergic Reactions to COVID-19 Vaccines

**DOI:** 10.3390/vaccines13030289

**Published:** 2025-03-10

**Authors:** Karnsinee Thanborisutkul, Prapasri Kulalert, Kanthida Methaset, Sira Nanthapisal, Tibet Chunthatikul, Nathamon Phangpanya, Phenpraphatson Charoenying, Worakamon Atsawutmangkru, Suphatsara Srijaroen, Patcharaporn Punyashthira, Orapan Poachanukoon

**Affiliations:** 1 Department of Pediatrics, Faculty of Medicine, Thammasat University, Pathum Thani 12120, Thailand; karnsinee.than@gmail.com (K.T.); nsira@tu.ac.th (S.N.); nanofang@gmail.com (P.P.); orapanpoachanukoon@yahoo.com (O.P.); 2 Center of Excellence for Allergy, Asthma and Pulmonary Disease, Thammasat University Hospital, Thammasat University, Pathum Thani 12120, Thailand; 3 Department of Clinical Epidemiology, Faculty of Medicine, Thammasat University, Pathum Thani 12120, Thailand; 4 Department of Pharmacy, Thammasat University Hospital, Thammasat University, Pathum Thani 12120, Thailand; m.kanthida058@gmail.com; 5 Doctor of Medicine Program, Faculty of Medicine, Thammasat University, Pathum Thani 12120, Thailand; tibetfuji@gmail.com (T.C.); nathamon2545@gmail.com (N.P.); plankplankton5@gmail.com (P.C.); worakamonjeen@gmail.com (W.A.); suphatsara.bs@gmail.com (S.S.)

**Keywords:** COVID-19 vaccine, safety, adverse events following immunization, CoronaVac, ChAdOx1

## Abstract

**Background**: Few reports exist regarding the incidence and factors associated with allergic reactions to COVID-19 vaccines during post-marketing surveillance, especially for inactivated whole virus or viral vector vaccines. We aimed to determine the incidence and factors associated with self-reported allergic reactions to COVID-19 vaccines in the Thai population. **Methods**: A cross-sectional case-control study was conducted via telephone-based interviews. Cases were defined as physician-confirmed, self-reported vaccine recipients diagnosed with non-severe immediate allergic reactions, anaphylaxis, or delayed allergic reactions. Controls were randomly sampled from vaccinated individuals who reported no adverse events and were matched by the type of vaccine (1 case:2 controls). Demographic information and the history of atopic diseases were collected in both groups. Conditional logistic regression analysis was used to explore associated factors. **Results**: Among 215,079 vaccine doses administered, the incidence of self-reported skin symptoms of allergic reactions was 1821 events (0.85%). The risk factors for allergic reactions included age < 60 years (aOR 3.53; 95% CI:1.43–8.70; *p* = 0.006), female sex (aOR 8.33; 95% CI: 4.35–15.94; *p* < 0.001), a personal history of allergic rhinitis (aOR 4.32; 95% CI: 2.43–7.69; *p* < 0.001), atopic dermatitis (aOR 4.27; 95% CI: 1.74–10.47; *p* = 0.002), food allergies (aOR 6.53; 95% CI: 2.42–17.61; *p* < 0.001), and a family history of allergic disease (aOR 2.14; 95% CI: 1.12–4.08; *p* = 0.021). **Conclusions**: COVID-19 vaccines showed a low incidence of self-reported allergic reactions, which were more likely to occur in younger individuals, females, and those with a history of atopic diseases.

## 1. Introduction

The outbreak of coronavirus disease 2019 (COVID-19) that emerged in Wuhan, China, was first identified in December 2019 [1]. It rapidly spread worldwide and became a global pandemic that has impacted health and global economies resulting in morbidity and mortality [2,3,4]. Currently, several types of COVID-19 vaccines are available that include inactivated vaccines (e.g., CoronaVac), mRNA vaccines, such as BNT162b2 (Pfizer BioNTech, London, UK) and mRNA-1273 (Moderna, Cambridge, MA, USA), and vector vaccines, such as ChAdOX1 nCOV-19 (Oxford-AstraZeneca, Cambridge, UK) [5]. They have shown efficacy in preventing COVID-19 infections [6,7,8,9].

In addition to efficacy, adverse events following immunization (AEFI) to the COVID-19 vaccines are an important issue associated with COVID-19 vaccine hesitancy related to patient preference and physician advice. From the clinical trials, the COVID-19 vaccines showed a safety profile with a low incidence of AEFI. Polack et al. [6] conducted a multinational, placebo-controlled, observer-blinded, efficacy and safety trial of the BNT162b2 vaccine and reported that local reactions were often reported in the BNT162b2 group. Mild-to-moderate pain at the injection site was the most common local reaction. Likewise, Baden et al. [7] conducted a randomized, observer-blinded, placebo-controlled trial for the efficacy and safety of the mRNA-1273 vaccine and showed that solicited local and systemic adverse events occurred more frequently in the mRNA-1273 group than in the placebo group. Serious adverse events were rare, and the incidence was similar in the two groups. Similarly, Falsey et al. [8] reported that AZD1222 was safe, with low incidences of serious and medically attended adverse events. The most common adverse events were general pain, headache, injection-site pain, and fatigue. Durusu et al. [9] reported that CoronaVac has a good safety and tolerability profile, fatigue was the most common systemic adverse event, and injection-site pain was the most frequent local adverse event, respectively.

Post-marketing surveillance is the practice of monitoring the safety of a pharmaceutical drug after its release on the market. During post-marketing surveillance from December 2020 through January 2021, the Centers for Disease Control and Prevention reported that the anaphylaxis cases following BNT162b2 and mRNA-1273 vaccinations had incidence rates of 4.7 and 2.5 cases per million doses, respectively [10]. From February to March 2021 in Japan, the Committee on Drug Safety of the Pharmaceutical Affairs and Food Sanitation Council and the Vaccine Adverse Reaction Review Committee evaluated cases of suspected event reports of anaphylaxis after the BNT162b2 vaccination. They reported an incidence rate of 8.1 cases per 100,000 doses of suspected vaccine event reports [11]. Several studies have reported that the factors associated with allergic reactions to the mRNA COVID-19 vaccines included a younger age, female, and personal history of allergic disease [12,13,14,15].

However, to the best of our knowledge, studies have not reported the incidences and factors associated with allergic reactions due to inactivated vaccines (e.g., CoronaVac) and vector vaccines, such as ChAdOX1 nCOV-19 (Oxford-AstraZeneca), during the post-marketing phase. Filling this gap in knowledge will help develop the surveillance and monitoring of COVID-19 recipients with risk factors.

The AZD1222 and CoronaVac vaccines were available in Thailand and administrated as the mainstay vaccines in the early period of the pandemic. Thammasat University Hospital is one of the mass vaccination centers in Thailand where more than 200,000 recipients received vaccines including CoronaVac, AZD1222, and BNT162b2 between 1 March and 30 September 2020. Therefore, we aimed to determine the incidence of self-reported AEFI and self-reported allergic reactions. We also explored the factors associated with self-reported allergic reactions to COVID-19 vaccines.

## 2. Materials and Methods

### 2.1. Study Design and Populations

A cross-sectional study was conducted to collect data on self-reported AEFI to COVID-19 vaccines from the Thai application database, which was developed by The Ministry of Public Health of Thailand. All COVID-19 recipients aged ≥ 12 years old who received the CoronaVac, AZD1222, or BNT162b2 COVID-19 vaccines at Thammasat University Hospital between 1 March and 30 September 2021, were enrolled.

All recipients who reported cutaneous symptoms were contacted via telephone by physicians to confirm the allergic reactions. The relevant clinical findings that included symptoms, onset of symptoms after vaccination, and data on medications received were also collected. Self-reported allergic reactions were classified as non-severe, immediate allergic reaction (NSIR) (e.g., urticaria and/or angioedema < 4 h), anaphylaxis, or delayed allergic reaction (DR) (e.g., urticaria ≥ 4 h or maculopapular rash) [16,17].

Then, we conducted a case-control study to explore the factors associated with self-reported allergic reactions to COVID-19 vaccines. Cases were defined as physician-confirmed self-reported vaccine recipients diagnosed as NSIR, anaphylaxis, or DR [16,17]. If a recipient showed the same type of allergic reaction at the first and second dose of vaccination, data for only the first dose were collected. If the recipients reported different types of allergic reactions at the first and second doses, the data of each dose were recorded. Controls were randomly sampled from those who reported no AEFI and matched with the case by the type of vaccine received (1 case:2 controls).

### 2.2. Data Collection

Both case and control groups were interviewed via telephone and online questionnaires for baseline characteristic collection that included age, gender, body weight, height, personal history of atopic disease (i.e., asthma, allergic rhinitis, atopic dermatitis, food allergy, and drug allergy), or family history of allergic disease.

Adverse events to COVID-19 vaccines were defined based on safety assessments for the clinical development of COVID-19 vaccines licensed by in the US FDA. COVID-19 vaccine adverse events were classified as the following: (1) solicited adverse events that were predictable following immunization and the reactogenicity of the vaccine reported by the vaccine recipient within 7 days after vaccination, and the reactions were divided into local and systemic reactions; (2) unsolicited adverse events that were unpredictable and reported by the vaccine recipients or healthcare workers within 28 days after vaccination; (3) medically attended adverse events; (4) serious adverse events that were life-threatening adverse events that required hospitalization and caused disability or a substantial disruption of the ability to conduct normal life function or death [18].

Allergic reactions were classified into three types based on the characteristics of cutaneous symptoms and the timing of onset after vaccination as follows: (1) non-severe, immediate allergic reactions (NSIRs) included acute onset urticaria or angioedema occurring within 4 h of vaccination [16]. Urticaria was characterized by the sudden appearance of wheals with circumscribed erythema and edema of the superficial dermis in variable numbers and sizes, with or without angioedema. Angioedema involved edema in the deeper dermis and subcutis, often affecting the face, as well as the buccal mucosa, tongue, larynx, and pharynx [17]. (2) Anaphylaxis was defined as the acute onset of urticaria or angioedema accompanied by systemic involvement, such as cardiovascular, respiratory, or gastrointestinal symptoms following vaccination. Confirmatory anaphylaxis was diagnosed based on the Brighton Collaboration definition [19]. (3) Delayed allergic reactions (DRs) included the delayed onset of urticaria or angioedema occurring more than 4 h after vaccination [16], or maculopapular eruptions occurring within 7 days following vaccination. Maculopapular exanthem was characterized by macular or papular rashes, or both, accompanied by pruritus without other systemic involvement [17].

### 2.3. Study Variables

Candidate factors associated with self-reported allergic reactions to COVID-19 vaccines were identified from the literature review. Previous studies reported a younger age [12,20], female [12,13], and personal history of atopic disease [12,13,14,15] as potential risk factors for allergic reactions to COVID-19 vaccines. Also included were a personal history of drug or vaccine allergy [21], family history of allergic disease in first-degree relatives [22], increased risk of drug allergy. Therefore, we selected these risk factors as interesting variables.

### 2.4. Statistical Analysis

The incidence of self-reported allergic reactions to vaccines used the number of COVID-19 vaccine recipients who reported allergic reactions between 1 March and 30 September 2021, at our center as the numerator and the total number of vaccine recipients within the same period as the denominator. The frequency and percentage described the categorical variables, and the mean and standard deviation or median and interquartile range (IQR) were used to describe continuous variables as appropriate. Categorical data were analyzed based on Fisher’s exact test. An independent t-test was used for continuous data with a normal distribution, and the Wilcoxon rank sum test was used for skewed data. A two-sided *p*-value < 0.05 was considered statistically significant. Conditional logistic regression was used to explore the factors associated with allergic reactions. Variables that were potential risk factors associated with allergic reactions to COVID-19 vaccines, as observed via univariable analysis (*p* < 0.05), were further processed through multivariable analysis and are presented as the adjusted odds ratio (aOR) with the 95% confidence interval (CI). All statistical analyses were performed using Stata version 17 (StataCorp, Lakeway, TX, USA).

## 3. Results

### 3.1. Incidence of Self-Reported AEFI

**Figure 1:**
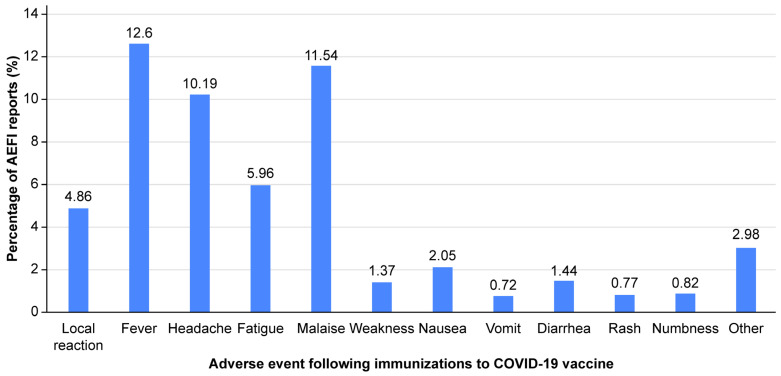
Self-reported solicited adverse events following all COVID-19 vaccinations at Thammasat University Hospital between 1 March and 30 September 2021.

**Figure 2:**
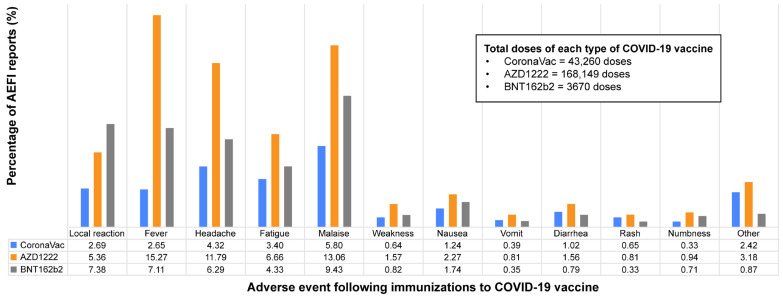
Self-reported solicited adverse events following immunization by type of COVID-19 vaccination at Thammasat University Hospital between 1 March and 30 September 2021.

A total of 215,079 doses of COVID-19 vaccines were administered to Thai people at Thammasat University Hospital between 1 March and 30 September 2021. Fifty-three thousand and one hundred self-reported SAEs were reported via the application within 7 days after vaccination. The overall incidence rate of self-reported SAEs to COVID-19 vaccines was 24.69%. All self-reported SAEs following all types of vaccine are shown (Figure 1). The three most common AEFIs were fever, malaise, and headache in descending order. Of the 43,260 doses of CoronaVac, the three most common AEFIs were malaise, headache, and fatigue in descending order. In the AZD1222 group with 168,149 vaccine doses, the three most common AEFIs were fever, malaise, and headache. Of the 3670 doses of the BNT162b2 vaccine, the three most common AEFIs were malaise, local reaction, and fever (Figure 2).

### 3.2. Incidence of Self-Reported Allergic Reactions

**Figure 3:**
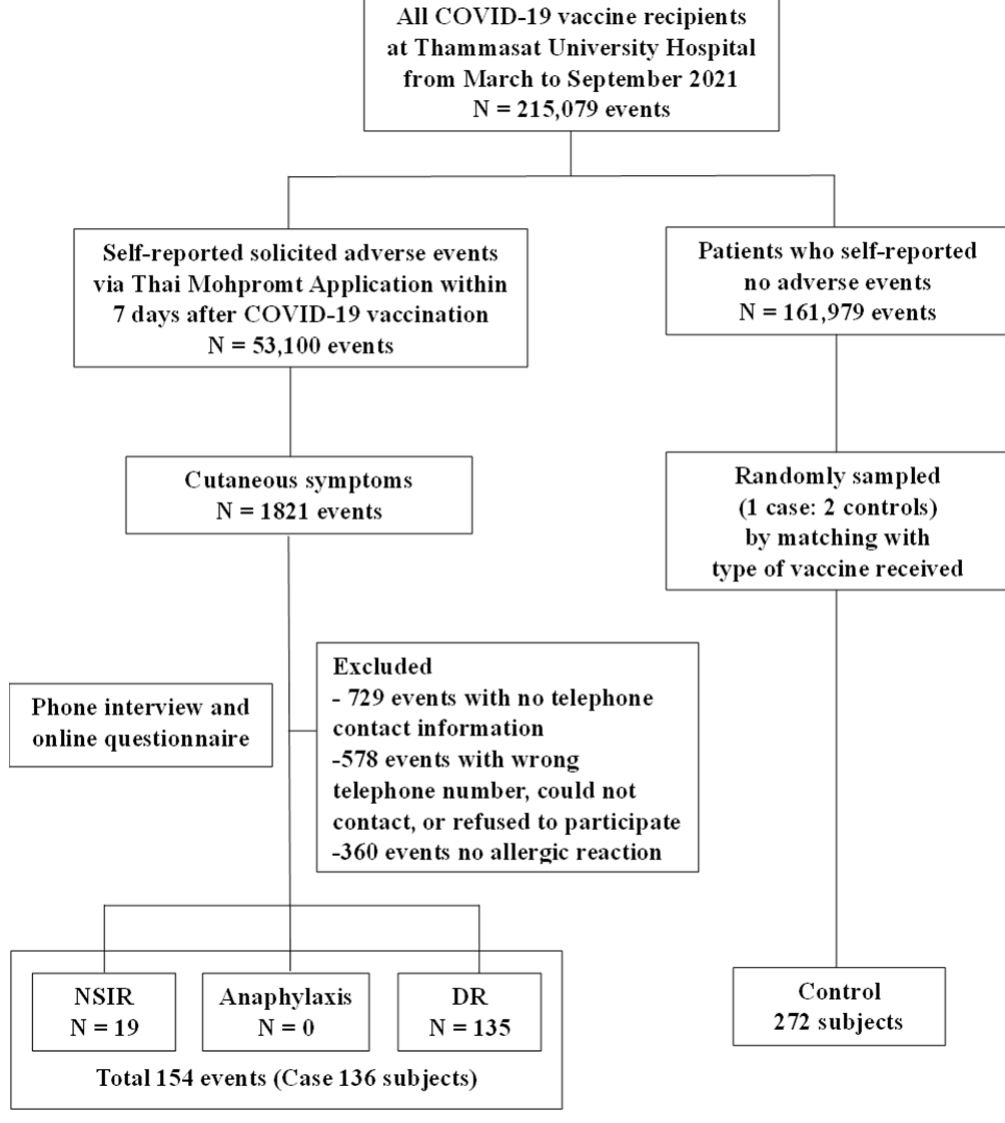
Flow diagram of all COVID-19 vaccine recipients at Thammasat University Hospital between 1 March and 30 September 2021. NSIR non-severe, immediate allergic reaction; DR delayed allergic reaction.

During the study period, a total of 1821 events of self-reported cutaneous symptoms were reported via the application. The incidence rate of self-reported skin symptoms of allergic reactions to COVID-19 vaccines was 846.67 (0.85%) per 100,000 injections. A total of 1821 events of self-reported cutaneous symptoms were reported via the application, and of the 1307 events, 729 events were excluded due to no telephone contact information, and 578 events were excluded due to wrong telephone numbers, could not contact, or the recipients refused to participate. Five hundred and fourteen events were interviewed and confirmed as allergic reactions by physicians. However, 360 events were not enrolled due to no allergic reaction after confirmation by a physician. Finally, we confirmed 154 events from 136 cases as allergic reactions that included 19 (12.33%) NSIRs and 135 (87.66%) DRs. Anaphylaxis was not reported in this study (Figure 3).

### 3.3. Baseline Characteristics of the Participants

A total of 408 participants, including cases and controls (1 case:2 controls), were enrolled (136 cases and 272 controls). Among both groups, the most common type of administered vaccine was AZD1222. The median (IQR) ages in the case and control groups were 36 (28, 49) and 45 (31, 56) years, respectively, which were significantly different (*p* < 0.001), and females predominated in the case group (*p* < 0.001). Furthermore, a family history of allergic disease in first-degree relatives and a personal history of all allergic diseases, except asthma, were also statistically significantly different (*p* < 0.001) (Table 1).

### 3.4. Risk Factors for Self-Reported Skin Symptoms of Allergic Reactions to COVID-19 Vaccines

In the univariable analysis, recipients with allergic reactions to COVID-19 vaccines were significantly younger than 60 years (*p* < 0.001), female (*p* < 0.001) and had histories of allergic rhinitis (*p* < 0.001), atopic dermatitis (*p* < 0.001), and food allergy (*p* < 0.001). Furthermore, the vaccine recipients with allergic reactions had a previous vaccine or drug allergy (*p* = 0.021) and had a family history of allergic disease in first-degree relatives (*p* < 0.001). All observed variables were potentially associated with allergic reactions to COVID-19 vaccines (Table 2).

In the multivariable conditional logistic regression analysis, the significant variables associated with allergic reactions included age < 60 years (aOR 3.53; 95% CI: 1.43–8.70; *p* = 0.006), female (aOR 8.33; 95% CI: 4.35–15.94; *p* < 0.001), allergic rhinitis (aOR 4.32; 95% CI: 2.43–7.69; *p* < 0.001), atopic dermatitis (aOR 4.27; 95% CI: 1.74–10.47; *p* = 0.002), food allergy (aOR 6.53; 95% CI: 2.42–17.61; *p* < 0.001), and had a family history of allergic disease (aOR 2.14; 95% CI: 1.12–4.08; *p* = 0.021) (Table 3).

## 4. Discussion

This is the first population-based study conducted during the post-marketing surveillance of COVID-19 vaccines in the Thai population and included three types of vaccines (BNT162b2, AZD1222, and CoronaVac). Our study revealed a low incidence rate of self-reported allergic reactions to COVID-19 vaccines, and anaphylactic reactions were not reported, which was similar to the estimated incidence rate reported previously. Imai et al. [13] recruited 614,151 mRNA-1723 vaccine recipients from a mass vaccination center in Japan and reported low incidence rates of immediate hypersensitivity reaction (IHSR) and anaphylaxis. The incidence rate of IHSR per million doses was 266 cases, and anaphylaxis was 2 cases, respectively. Macy et al. also conducted a retrospective review of electronic health records, and there were only 2 of 606,273 (0.00033%) vaccinations, and they reported that the population-based incidence of confirming anaphylaxis was 3.29 per million vaccine doses [23].

In contrast, Blumenthal et al. [24] conducted a questionnaire-based study, including 64,900 healthcare employees at Mass General Brigham. They reported that the incidence of allergic symptoms after the first dose of mRNA COVID-19 vaccination was 2.1%, and anaphylactic reactions occurred at a rate of 2.47 per 10,000 injections. Shavit et al. [14] prospectively performed a study in Israel and documented rates of allergic reactions of 1.4% and anaphylaxis of 0.7% in 429 highly allergic patients who received BNT162b2 vaccines. The incidence rates of allergic reactions in our study were significantly lower than those reported in those studies, which was possibly due to different methods among the studies. In addition to the self-reported allergic reactions of our participants, we confirmed the reactions through telephone follow-up by physicians to increase the reliability of the allergic reaction evaluation.

Currently, the known factors associated with an increased risk of allergic reactions to COVID-19 vaccines include a younger age [12,20,23], female [12,13,23,25], and a personal history of allergic disease [21,23,25]. However, these findings explored associating factors with allergic reactions to mRNA COVID-19 vaccines. We explored the factors associated with self-reported allergic reactions to COVID-19 vaccines, including viral vector and inactivated vaccines. By comparing clinical characteristics in this study between recipients with and without reported allergic reactions, we identified four factors associated with self-reported allergic reactions to COVID-19 vaccines: (1) younger age (<60 years); (2) female; (3) personal history of allergic disease (i.e., allergic rhinitis, atopic dermatitis, food allergy); and (4) family history of allergic disease in first-degree relatives.

Of the four factors, being female was the most potent predictor of the development of allergic reactions following COVID-19 vaccination (OR 8.33; 95% CI: 4.35–15.94; *p* < 0.001). This was in concordance with previous studies. Iguchi et al. [11] and Bian et al. [12] reported that anaphylaxis following mRNA vaccination was more commonly observed in females than males. Likewise, Imai et al. [13] documented that female recipients were associated with IHSR after mRNA-1723 administration (OR 3.69; 95% CI: 2.56–5.31; *p* < 0.001). Being female is also a factor that can predict drug hypersensitivity. Although many reports showed that being female was a significant risk factor for allergic reactions to the COVID-19 vaccine and other drugs, to date, no known pathophysiological evidence has been reported.

Many studies also reported that a younger age was a predictor of allergic reactions to COVID-19 vaccines; however, differences existed in the cut-off ages in these studies. Bian et al. [12] documented that those recipients who reported allergic reactions had a mean age of 45.11 ± 5.6 years vs. 47.01 ± 6.3 years (*p* < 0.001). Similarly, Paoletti et al. reported that immediate allergic reactions were more frequent in the younger age group [25]. In this current study, we used a cut-off age of 60 years, which is the same for the elderly in Thailand, defined as a high-risk group for COVID-19 infections and receiving priority for vaccination. Our results showed that a younger age was one factor associated with allergic reactions to COVID-19 vaccines (aOR 3.53; 95% CI: 1.43–8.70; *p* = 0.006).

A personal history of allergic disease was also an important factor that several reports and international guidelines for COVID-19 vaccine allergy management focused on. In this study, we found that allergic rhinitis, atopic dermatitis, and food allergy were related to allergic reactions to COVID-19 vaccines. Similarly, Iguchi et al. [11] and Bian et al. [12] reported that anaphylaxis following mRNA vaccination was more frequently observed in recipients with a prior history of allergy. Desai et al. [15] documented that a pre-existing history of allergy and anaphylaxis had a 2- and 7-times higher incidence rate of anaphylaxis after an mRNA vaccination compared with those without any history of allergy. Likewise, Imai et al. [13] reported that mRNA-1723 recipients who had a history of asthma, atopic dermatitis, and food allergy had a statistically significantly increased risk of developing IHSR. These factors were in concordance with most previous studies that mentioned mRNA COVID-19 vaccine recipients. Interestingly, for our study, most data were collected from the viral-vector-based vaccine and inactivated vaccine. We hypothesize that the predicting factors identified in this current study may be important factors for the prediction of all types of COVID-19 vaccine hypersensitivity reactions. Further studies are needed to provide more data.

A family history of allergy was described as a predictor of drug hypersensitivity. Previously, no data were available on COVID-19 vaccines. This current study is the first to show that a family history of allergic disease in first-degree relatives was also significantly associated with allergic reactions following vaccination (aOR 2.14; 95% CI: 1.12–4.08; *p* = 0.021).

Some clinically relevant factors have been reported to be associated with allergic reactions to COVID-19 vaccines but did not show statistical significance in our study. For example, it was reported that people with a history of drug allergy had a higher risk of developing allergic reactions following vaccination [21,23]. Similarly, Imai et al. [13] reported that allergic reactions to the mRNA-1723 vaccine were significantly reported by recipients with a prior history of drug allergy (OR 13.32; 95% CI: 7.57–23.44; *p* < 0.001). Even though a history of drug allergy was not statistically significant in our study, clinical practice should be aware of allergic reactions to COVID-19 vaccines in people with this risk.

Our findings provide the incidence rates of allergic reactions to COVID-19 vaccines that mostly reflect a viral vector-based vaccine and an inactivated vaccine, which differ from previously published studies. Moreover, we identified factors associated with allergic reactions after vaccination to increase the awareness of COVID-19 vaccination in people who have these risk factors.

This study has some strengths. First, it included a large population of more than 200,000 participants. Second, even though several studies on the incidence and factor-related COVID-19 vaccines allergic reactions are available, most of them focused on mRNA vaccines. This study differs from previous studies because it focused on the viral vector-based and inactivated COVID-19 vaccines. Third, this study was one of the few studies that collected data in an Asian country. However, this study also has some limitations. First, some parts of the study involved retrospective data collection, which may be subject to recall bias. Although we made efforts to minimize this bias by providing a questionnaire that included images of all types of cutaneous reactions to aid the participants’ memory, we acknowledge that it is impossible to completely eliminate the risk of recall bias and recognize that some degree of recall bias may still persist. Second, since our outcome was based on self-reported allergic reactions from vaccine recipients, there is a possibility that the incidence rate may have been overestimated. To increase the reliability of these self-reports, we confirmed the allergic reactions via telephone interviews conducted by physicians, using the clinical history, onset of reaction, and type of reaction. However, we acknowledge that despite this confirmation, self-reporting still remains a potential source of bias, and some mild reactions may not have been recorded.

## 5. Conclusions

COVID-19 vaccines showed a very low incidence rate of self-reported allergic reactions. Allergic reactions were more likely to occur in vaccine recipients who were <60 years old, female, and had a history of atopic disease. Our study suggests that people with these risk factors should be closely monitored after COVID-19 vaccination.

## Figures and Tables

**Table 1:** Baseline characteristics of the participants. NA = not available.

Variables	Case (n = 136)	Control (n = 272)	*p*-Value
Type of COVID-19 vaccine			
CoronaVac	21	42	NA
AZD1222	113	226	NA
BNT162b2	2	4	NA
Median (IQR) age, years	36 (28, 49)	45 (31, 56)	<0.001
Age group			
<60 years	128 (94.12)	216 (79.41)	<0.001
≥60 years	8 (5.88)	56 (20.59)	
Gender			
Female	117 (86.03)	127 (46.69)	<0.001
Male	19 (13.97)	145 (53.31)	
Median (IQR) BMI (kg/m^2^)	22.92 (20.57, 25.95)	23.44 (20.96, 26.32)	0.310
Obesity (BMI ≥ 30 kg/m^2^)	9 (6.62)	26 (9.56)	0.354
Personal history of allergic disease			
Allergic rhinitis	67 (49.26)	42 (15.44)	<0.001
Asthma	6 (4.41)	9 (3.31)	0.585
Atopic dermatitis	30 (22.06)	13 (4.78)	<0.001
Food allergy	28 (20.59)	9 (3.31)	<0.001
Vaccine/drug allergy	20 (14.71)	20 (7.35)	0.022
Family history of allergic disease (first-degree relatives)	43 (31.62)	29 (10.66)	<0.001

**Table 2:** Factors associated with self-reported skin symptoms of allergic reactions to COVID-19 vaccines using univariable conditional logistic regression.

Variables	Crude Odds Ratio	95% CI	*p*-Value
Age < 60 years	4.31	1.98–9.39	<0.001
Female gender	6.97	4.06–11.95	<0.001
Allergic rhinitis	5.40	3.36–8.68	<0.001
Atopic dermatitis	5.66	2.84–11.27	<0.001
Food allergy	7.43	3.40–16.22	<0.001
Vaccine/drug allergy	2.17	1.12–4.18	0.021
Family history of allergic disease	3.87	2.28–6.56	<0.001

**Table 3:** Factors associated with self-reported skin symptoms of allergic reactions to COVID-19 vaccines using multivariable conditional logistic regression.

Variables	Adjusted Odds Ratio	95% CI	*p*-Value
Age < 60 years	3.53	1.43–8.70	0.006
Female gender	8.33	4.35–15.94	<0.001
Allergic rhinitis	4.32	2.43–7.69	<0.001
Atopic dermatitis	4.27	1.74–10.47	0.002
Food allergy	6.53	2.42–17.61	<0.001
Vaccine/drug allergy	1.02	0.44–2.35	0.964
Family history of allergic disease	2.14	1.12–4.08	0.021

## Data Availability

Data will be made available on request.

## Abbreviations

The following abbreviations are used in this manuscript:

AEFI	Adverse events following immunization
NSIR	Non-severe, immediate allergic reaction
DR	Delayed allergic reaction
